# Pluripotent stem cell-derived neural progenitor cells can be used to model effects of IL-6 on human neurodevelopment

**DOI:** 10.1242/dmm.050306

**Published:** 2023-11-03

**Authors:** Kseniia Sarieva, Felix Hildebrand, Theresa Kagermeier, Zeynep Yentür, Katharina Becker, Simone Mayer

**Affiliations:** ^1^ Hertie Institute for Clinical Brain Research, University of Tübingen, 72076 Tübingen, Germany; ^2^ International Max Planck Research School, Graduate Training Centre of Neuroscience, University of Tübingen, 72076 Tübingen, Germany; ^3^ Heidelberg Academy of Sciences and Humanities, 69117 Heidelberg, Germany

**Keywords:** Maternal immune activation, Interleukin-6, Induced pluripotent stem cells, Neural progenitor cells

## Abstract

Maternal immune activation (MIA) increases the risks for neurodevelopmental disorders in offspring through inflammatory cytokines, including interleukin-6 (IL-6). We therefore aimed to establish a human two-dimensional (2D) *in vitro* neural model to investigate the effects of IL-6 exposure on neurodevelopment. IL-6 signal transduction requires two receptors: interleukin-6 signal transducer (IL6ST) and interleukin-6 receptor (IL6R). Prenatally, neural cells lack IL6R, and hence cannot elicit *cis* IL-6 signaling, but IL6R can be provided by microglia in *trans*. We demonstrate here that an immortalized human neural progenitor cell (NPC) line, ReNCell CX, expresses IL6ST and elicits both *cis* and *trans* IL-6 signaling, limiting its use as a model of MIA. In contrast, induced pluripotent stem cell (iPSC)-derived NPCs only activate the IL-6 cascade in *trans*. Activation of the *trans* IL-6 cascade did not result in increased proliferation of iPSC-derived NPCs or ReNCell CX, as has been demonstrated in animal models. iPSC-derived NPCs upregulated NR2F1 expression in response to IL-6 signaling in line with analogous experiments in organoids. Thus, iPSC-derived NPCs can be used to model gene expression changes in response to MIA in 2D cultures.

## Teaser

**Summary:** We characterize how two distinct two-dimensional neural progenitor cell cultures respond to treatment with the cytokine interleukin-6 to model the effects of maternal immune activation on human neurodevelopment.

## INTRODUCTION

Maternal immune activation (MIA) during critical periods of prenatal development has a long-established contribution to increased risks for neurodevelopmental disorders in offspring, including autism spectrum disorder (ASD) and schizophrenia (Brown, 2012; Atladóttir et al., 2010; Han et al., 2021). Epidemiological and rodent studies identified the molecular players that translate MIA to altered neurodevelopmental outcomes (Atladóttir et al., 2010; Lombardo et al., 2018; Smith et al., 2007; Meyer et al., 2006; Ben-Reuven and Reiner, 2021; Kalish et al., 2021). These include cytokines such as interleukin (IL)-6, IL-17 and others (Kalish et al., 2021; Choi et al., 2016; Ben-Reuven and Reiner, 2021; Smith et al., 2007). In rodents, MIA is modeled by injecting either lipopolysaccharide or polyinosinic:polycytidylic acid into the pregnant dam, mimicking bacterial and viral infection, respectively (Massrali et al., 2022). Animals serve as model systems, in which several levels, from behavioral to cellular and molecular, can be analyzed (Kalish et al., 2021). However, in animal models, an array of inflammatory mediators is released in response to the treatment of the pregnant dam, precluding the analysis of the effects of individual cytokines on cell biology. Targeted analysis of specific inflammatory mediators are restricted to *in vitro* models, including stable cell lines of neural cells, primary cells derived from rodent brains, and induced pluripotent stem cell (iPSC)-derived two-dimensional (2D) and three-dimensional (3D) model systems (Kwan Cheung et al., 2022; Johansson et al., 2008; Mirabella et al., 2021; Warre-Cornish et al., 2020; Couch et al., 2023; Sarieva et al., 2023).

ReNCell CX neural progenitor cells (NPCs), a stable neural cell line, have been used to investigate mechanisms of action of different compounds (Kwan Cheung et al., 2022; Zhao et al., 2013). ReNCell CX cells were derived from human fetal neocortex and transduced with c-Myc (*MYC*) oncogene for immortalization (Donato et al., 2007). The obtained immortalized human NPCs grow in a monolayer for extended periods of time and are easy to handle. An important feature of ReNCell CX cells is that, upon withdrawal of the stemness maintenance factors EGF and FGF2, they differentiate to a mixed culture of neural cells, including neurons, astroglial cells and oligodendroglial cells, with neurons never reaching functional maturation (Donato et al., 2007; Oh et al., 2017).

iPSC-derived neural cells are extensively used in developmental neuroscience as an *in vitro* model for cellular neurodevelopment (Ardhanareeswaran et al., 2017; Linda et al., 2018). With relevance to MIA, iPSC-derived NPCs and neurons were used to investigate the cellular and molecular effects of IFN-γ (Warre-Cornish et al., 2020), IL-1β (Park et al., 2018) and TNF-α (TNF) (Lee et al., 2020) in 2D or adherent cultures. Another advancement of iPSC-derived models was brain organoids, 3D cultures that recapitulate some key aspects of human neurodevelopment, including cellular composition and cytoarchitecture (Lancaster et al., 2013; Kadoshima et al., 2013). Compared to brain organoids, 2D cultures are characterized by increased reproducibility of differentiation protocols, relative easiness of genetic manipulation and handling, and scalability (Lovett et al., 2020). The major drawback of the 2D models compared to brain organoids are the lack of cellular microenvironment and close cell–cell interactions (Lovett et al., 2020; Tian et al., 2020). Finally, both 2D and 3D iPSC-derived neural models suffer from the variability of the differentiation potential between the iPSC lines (Kanton et al., 2019; Shi et al., 2017).

In animal studies, IL-6 was causal of behavioral abnormalities upon MIA induction (Smith et al., 2007). At the cellular level, IL-6 influences the proliferation of NPCs in the developing mouse brain (Ben-Reuven and Reiner, 2021). This results in an increased number of deep-layer neocortical neurons, suggesting that IL-6 impairs the balance between self-renewal and differentiation (Ben-Reuven and Reiner, 2021). IL-6 acts through its receptor molecules, interleukin-6 receptor (IL6R) and interleukin-6 signal transducer (IL6ST), on the cell surface to induce intracellular signaling via Janus kinase (JAK) and signal transducer and activator of transcription 3 (STAT3) (Rose-John, 2018). In classic, or *cis*-, IL-6 signaling, both IL6R and IL6ST are expressed in the target cell, whereas in *trans*-signaling, soluble IL6R is provided from neighboring cells. In the developing human brain, IL6R is expressed exclusively in microglia (Nowakowski et al., 2017), and there is increasing evidence that microglia can provide soluble IL6R (s-IL6R) to other brain cells, enabling *trans*-signaling (Couch et al., 2023). Consistently, a recent study showed that iPSC-derived NPCs were not able to activate JAK/STAT3 signaling upon IL-6 treatment (Couch et al., 2023). Therefore, to model specifically *trans*-signaling in neural cellular systems devoid of microglia one should provide s-IL6R. This can be achieved by applying Hyper-IL-6, a synthetic protein consisting of s-IL6R and IL-6 linked through a flexible peptide chain (Rakemann et al., 1999; Sarieva et al., 2023).

Here, we aimed to establish an *in vitro* 2D model for investigating the cellular and molecular effects of IL-6 treatment as a tool to investigate the molecular consequences of IL-6 signaling in a scalable, reproducible manner. To achieve this, we used a stable line of NPCs, ReNCell CX, and iPSC-derived NPCs. We investigated the expression of IL6ST in these cellular model systems, as well as the activation of the downstream JAK/STAT pathway and their proliferative behavior. ReNCell CX cells demonstrated IL6ST expression and upregulated the level of phosphorylated (p)-Y705-STAT3 in response to both IL-6 and Hyper-IL-6, which is not reminiscent of the developing human neocortex. The iPSC-derived NPCs did not respond to IL-6 via *cis*-signaling in a previous study (Couch et al., 2023), and here we show that they can respond to Hyper-IL-6 through *trans*-signaling, indicating that they recapitulate the physiology of the developing human neocortex. The previously reported pro-proliferative properties of IL-6 signaling in NPCs were not observed in either of the cellular systems, highlighting the limitations of using 2D models to study cellular properties *in vitro*.

## RESULTS

### ReNCell CX cells as a model system for investigating the effects of IL-6 on human neurodevelopment

**Fig. 1.**
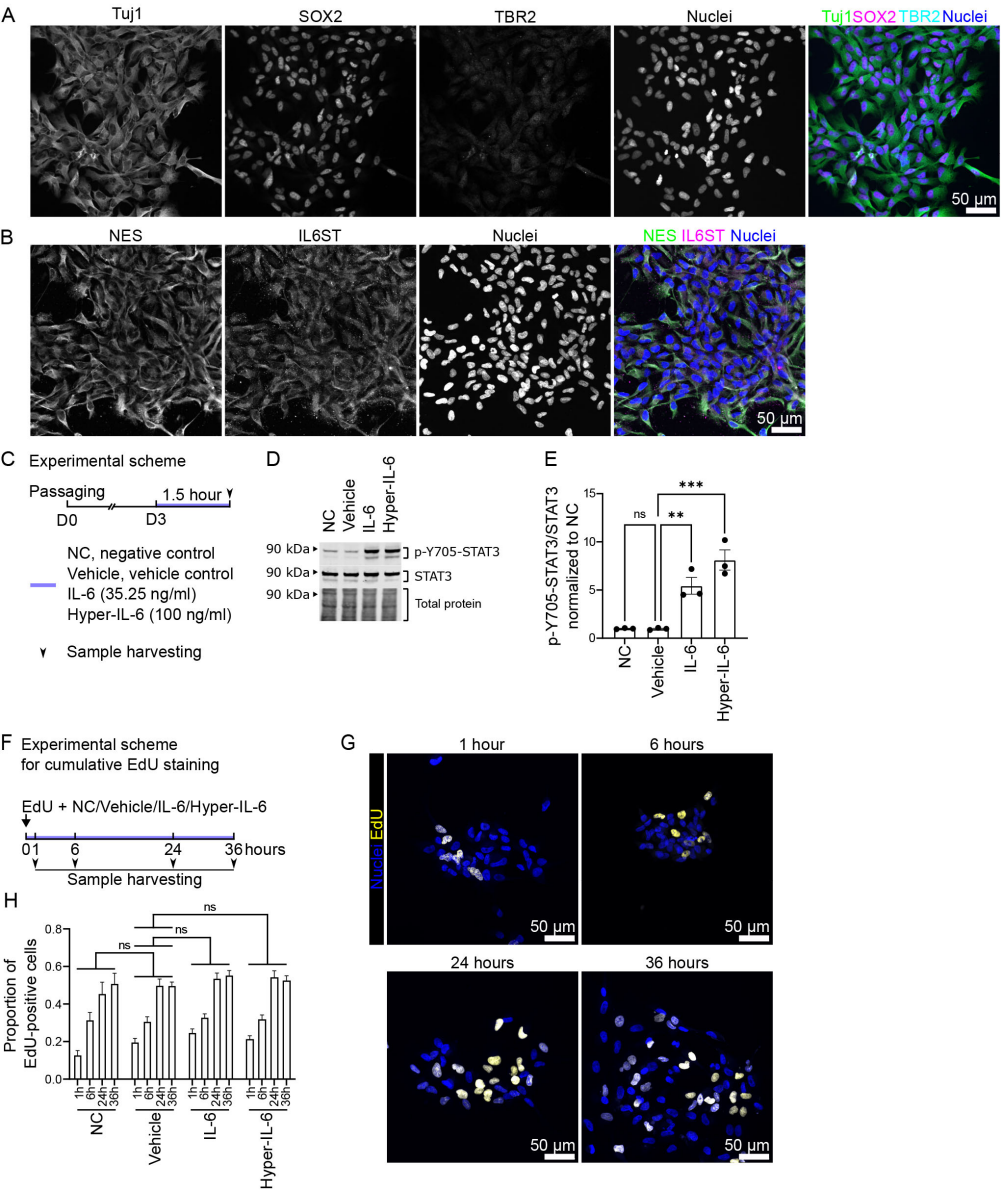
**ReNCell CX cells as a model for the effects of IL-6 exposure on neural progenitor cells.** (A) In ReNCell CX cell culture, SOX2-positive NPCs are also positive for Tuj1, a marker of young neurons, and negative for TBR2, a marker of intermediate progenitor cells (IPCs). Green, Tuj1; magenta, SOX2; cyan, TBR2; blue, nuclei. Scale bar: 50 µm. (B) In ReNCell CX cell cultures, IL6ST is expressed in NES-positive cells. Green, NES; magenta, IL6ST; blue, nuclei. Scale bar: 50 µm. (C) Scheme of the cytokine treatment in ReNCell CX cells. NC, negative control (PBS); Vehicle, vehicle control (0.1% BSA in PBS); IL-6, 35.25 ng/ml in 0.1% BSA in PBS; Hyper-IL-6, 25 ng/ml in 0.1% BSA in PBS. (D) Representative western blot of p-Y705-STAT3 (top), STAT3 (middle) and total protein (bottom) in ReNCell CX cells after treatment with IL-6 and Hyper-IL-6 as well as with NC and vehicle control. (E) Quantification of signal intensity of p-Y705-STAT3 relative to total STAT3 from D. Each dot represents an individual protein sample from consecutive passages of ReNCell CX cell culture (*n*=3 for each condition). Data are mean±s.e.m. Comparisons were analyzed using two-way ANOVA (passage×condition) followed by Dunnett's post-hoc test, comparing all other conditions to vehicle control: not significant (ns), *P*≥0.05, **P<0.01; ***P<0.001. (F) Experimental scheme for cumulative EdU treatment in ReNCell CX cells. NC, negative control (PBS); Vehicle, vehicle control (0.1% BSA in PBS); IL-6, 35.25 ng/ml in 0.1% BSA in PBS; Hyper-IL-6, 25 ng/ml in 0.1% BSA in PBS. (G) Representative images of EdU staining in vehicle-treated ReNCell CX cells over 1, 6, 24 and 36 h of exposure. Yellow, EdU; blue, nuclei. Scale bars: 50 µm. (H) Quantification of proportion of EdU-positive cells over total cell count from G (*n*=3 for each condition). Data are mean±s.e.m. Comparisons were analyzed using two-way ANOVA (length of exposure×condition) followed by Dunnett's post-hoc test, comparing all other conditions to vehicle control: ns, *P*≥0.05.

Here, we aimed to analyze the properties of different *in vitro* systems upon IL-6-depending signaling. Among human *in vitro* 2D systems, the ReNCell CX cells were proposed as a platform for testing chemical compounds in human NPCs (Donato et al., 2007; Breier et al., 2008). In line with previous reports (Donato et al., 2007; Lee et al., 2014), ReNCell CX cells were positive for both SOX2 and NES, nuclear and cytoplasmic markers of NPCs, respectively (Fig. 1A,B). Additionally, they were positive for class III beta-tubulin (Tuj1; TUBB3), which is a canonical marker of young neurons (Fig. 1A). Hence, ReNCell CX cells were positive for both NPC and neuronal markers. This phenomenon was previously reported (Oh et al., 2017). There were no cells positive for TBR2 (EOMES), a marker of intermediate progenitor cells (IPCs) of the neocortex (Fig. 1A).

To investigate whether we can use ReNCell CX cells to study the molecular effects of IL-6, we first analyzed the expression of the receptor IL6ST (Fig. 1B). We found that most cells expressed IL6ST. Second, we analyzed whether ReNCell CX cells were able to activate the canonical JAK/STAT signaling cascade upon acute IL-6/Hyper-IL-6 exposure for 1.5 h (Fig. 1C,D). The acute treatment paradigm was chosen because ReNCell CX cells do not differentiate in the presence of stemness maintenance factors. ReNCell CX cells showed increased phosphorylation of STAT3 at Y705 upon IL-6 (*P*=0.0036) and Hyper-IL-6 (*P*=0.0003) exposure compared to vehicle-treated controls (Fig. 1E) (Donato et al., 2007). Therefore, we conclude that ReNCell CX cells can activate both *cis* and *trans* IL-6 signaling. Mouse models of MIA suggest that IL-6 signaling induces proliferation of neocortical NPCs (Ben-Reuven and Reiner, 2021), and a similar phenotype is observed upon treatment of brain organoids with Hyper-IL-6 (Sarieva et al., 2023). We, therefore, aimed to analyze whether IL-6 and Hyper-IL-6 were able to induce proliferation in ReNCell CX cells. ReNCell CX cells were exposed to IL-6/Hyper-IL-6 and 5-ethynyl-2′-deoxyuridine (EdU) for 1, 6, 24 and 36 h, and the proportion of EdU-positive nuclei at each time point was analyzed (Fig. 1F). Using this approach, we found no changes in proliferation compared to vehicle-treated controls (*P*>0.05, Fig. 1G,H). Together, these results demonstrate that ReNCell CX cells can activate IL-6 signaling with both *cis* and *trans* mechanisms, but the activation of this signaling pathway does not lead to increased proliferation, thus not mimicking the cellular consequences reported in animal models (Ben-Reuven and Reiner, 2021).

### iPSCs efficiently differentiate into NPCs and develop susceptibility to Hyper-IL-6 treatment

**Fig. 2.**
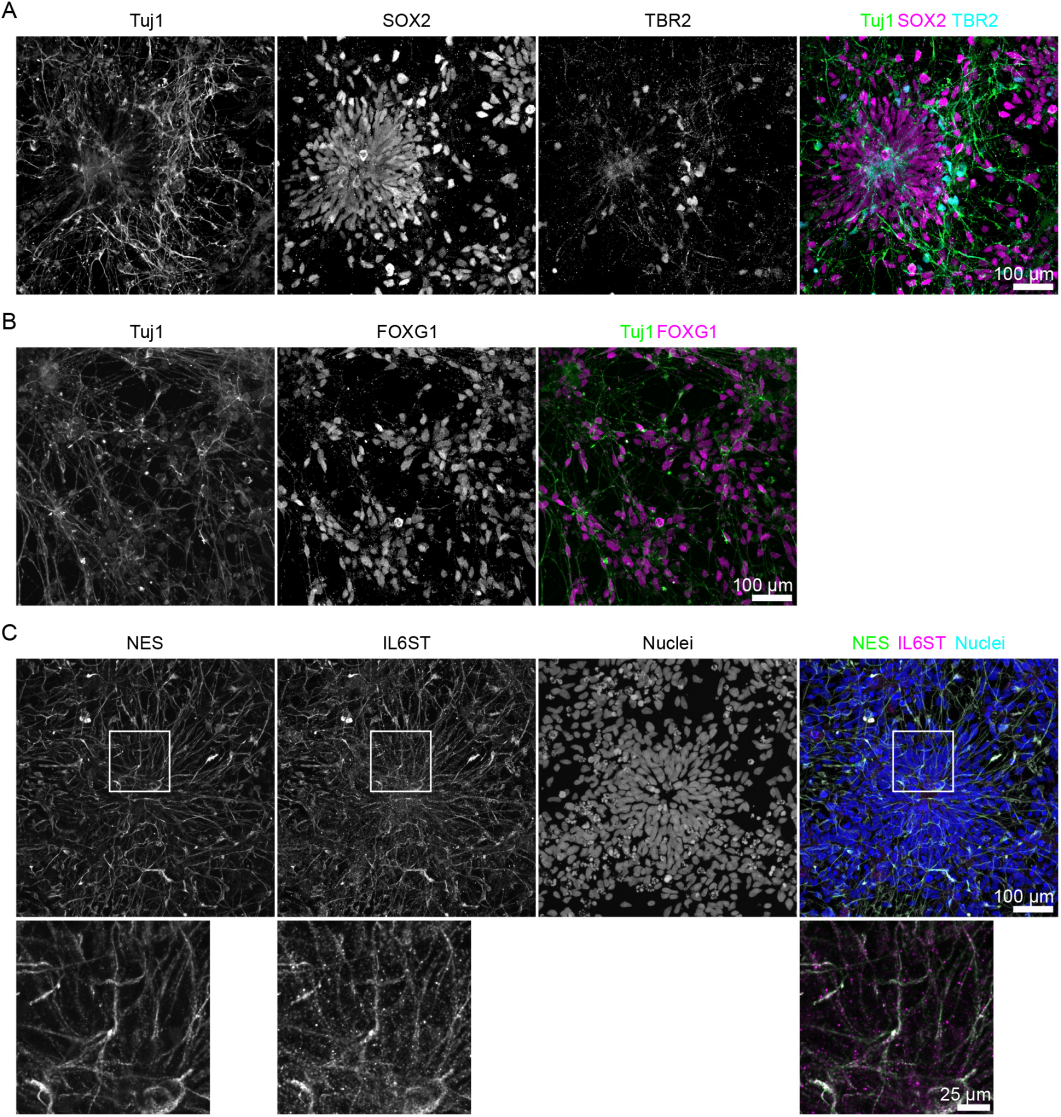
**Induced pluripotent stem cell (iPSC)-derived neural progenitor cells (NPCs) demonstrate forebrain regional identity and express IL6ST in NES-positive NPCs.** (A) In iPSC-derived NPCs, SOX2-positive cells are organized into rosettes with TBR2-positive intermediate progenitor cells located on the outer borders of the rosettes. Tuj1, a marker of young neurons, can primarily be found outside the rosette regions. Green, Tuj1; magenta, SOX2; cyan, TBR2. Scale bar: 100 µm. (B) Forebrain identity of NPCs was confirmed by the ubiquitous expression of FOXG1 in the culture. Green, Tuj1; cyan, FOXG1. Scale bar: 100 µm. (C) In the iPSC-derived NPCs cultures, IL6ST is associated with NES, the cytoplasmic marker of NPCs. Green, NES; magenta, IL6ST; blue, nuclei. Boxed areas are shown at higher magnification in the row below. Scale bars: 100 µm (top), 25 µm (bottom). Images represent maximum-intensity projections of *z*-stacks sampled in 1 µm steps. Images were subjected to ‘Despeckle’ function in ImageJ for visualization purposes.

While c-Myc expression likely changes ReNCell CX cells such that they fail to physiologically respond to IL-6 signaling, iPSC-derived neural cultures may serve as an alternative approach to study the molecular consequences of MIA induction *in vitro*. To test this hypothesis, we used a well-established protocol for the differentiation of iPSCs towards neocortical neurons (Shi et al., 2012) using an iPSC line with a described differentiation potential toward neural lineage (Rasmussen et al., 2014). We confirmed the differentiation towards the neural lineage by immunocytochemistry for the key markers for each cell type. On day (D)26 of differentiation, the cultures contained cells expressing NPC marker, SOX2, as well as the IPC marker, TBR2, and the marker of young neurons, Tuj1 (Fig. 2A). Forebrain specificity was confirmed by expression of FOXG1 (Fig. 2B). As reported previously, SOX2-positive cells were organized in rosettes reminiscent of the ventricular zone of the developing human brain (Fig. 2A) (Shi et al., 2012). Compared to ReNCell CX cells, in the iPSC-derived neural cultures, Tuj1 was largely excluded from the SOX2-positive areas, demonstrating separation between NPCs and neurons. TBR2-positive cells were found primarily on the outer edge of the rosettes, recapitulating the position of IPCs in the subventricular zone in the developing human neocortex (Fig. 2A). At this time point, cells expressed IL6ST, which was associated with NES, a cytosolic marker of NPCs (Fig. 2C), in line with their expression in radial glial cells in brain organoids (Sarieva et al., 2023). Corroborating previous findings (Couch et al., 2023), these data indicate that iPSC-derived neural cultures are likely to be only responsive to IL-6 exposure if s-IL6R is provided in *trans*.

### iPSC-derived neural cultures phosphorylate STAT3 at Y705 in response to Hyper-IL-6

**Fig. 3.**
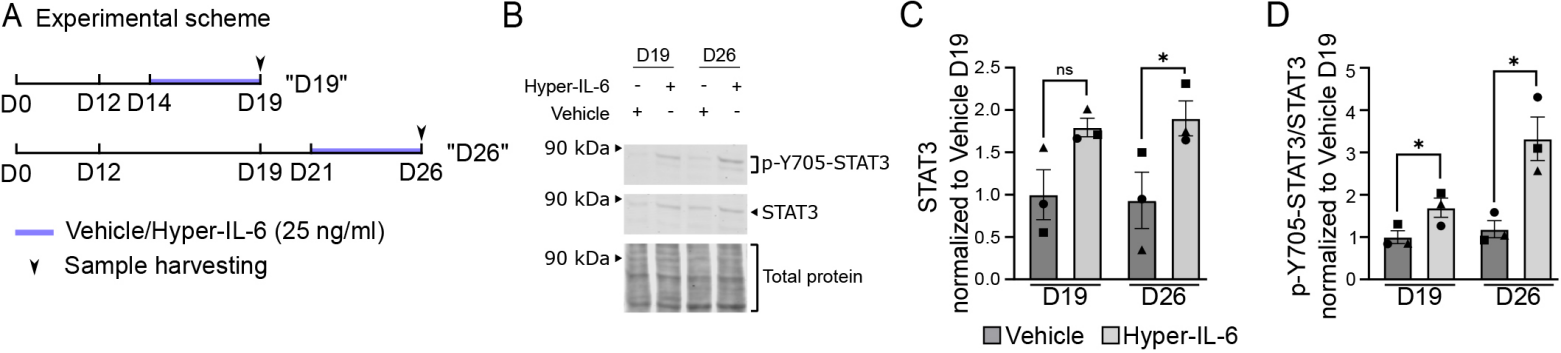
**Hyper-IL-6 treatment leads to the activation of the JAK/STAT intracellular signaling cascade in iPSC-derived NPCs.** (A) Scheme of the cytokine treatment in iPSC-derived NPCs. Vehicle, vehicle control (0.1% BSA in PBS); Hyper-IL-6, 25 ng/ml in 0.1% BSA in PBS. (B) Representative western blot of p-Y705-STAT3 (top), STAT3 (middle) and total protein (bottom) in iPSC-derived NPCs after 5 days of treatment with Hyper-IL-6 as well as with vehicle. (C) Quantification of signal intensity of total STAT3 relative to total protein stain from B. (D) Quantification of signal intensity of p-Y705-STAT3 relative to total STAT3 from B. For C and D, each dot represents an individual protein sample from consecutive differentiation batches of iPSC-derived NPCs (*n*=3 batches). Data are mean±s.e.m. Comparisons were analyzed using paired two-tailed Student's *t*-test separately for day (D)19 and D26: ns, *P*≥0.05; *P<0.05.

To assess the ability of iPSC-derived neural cultures to activate JAK/STAT signaling through the *trans* mechanism, we exposed the cells to 25 ng/ml Hyper-IL-6 over 5 days during two time periods, D14-D19 (D19) and D21-D26 (D26) of differentiation (Fig. 3A). This chronic exposure paradigm was chosen to mimic the persistently high levels of inflammatory cytokines during an infection. The concentration of Hyper-IL-6 roughly corresponds to the serum IL-6 concentration upon septic infection (Qiu et al., 2021). The two time periods were chosen to model different phases of development, specifically, the first one corresponding to a stage with predominant self-renewal and the second to a shift towards neuronal differentiation. Consistently, at both endpoints, D19 and D26, the cultures were dominated by the NPCs, with young neurons emerging at D26 (Fig. 2A,B). In response to Hyper-IL-6, the cells upregulated the absolute quantity of STAT3 at D26 (Fig. 3B,C, *P*=0.0467) and relative phosphorylation of STAT3 at Y705 (Fig. 3B,D) at both D19 (*P*=0.0422) and D26 (*P*=0.0250) compared to vehicle-treated controls. Together, these results demonstrate that iPSC-derived NPCs respond to IL-6 signaling through the *trans* mechanism.

### Hyper-IL-6 does not affect the proliferative capacity of iPSC-derived NPCs but reproduces gene expression changes observed in an organoid model of MIA

**Fig. 4.**
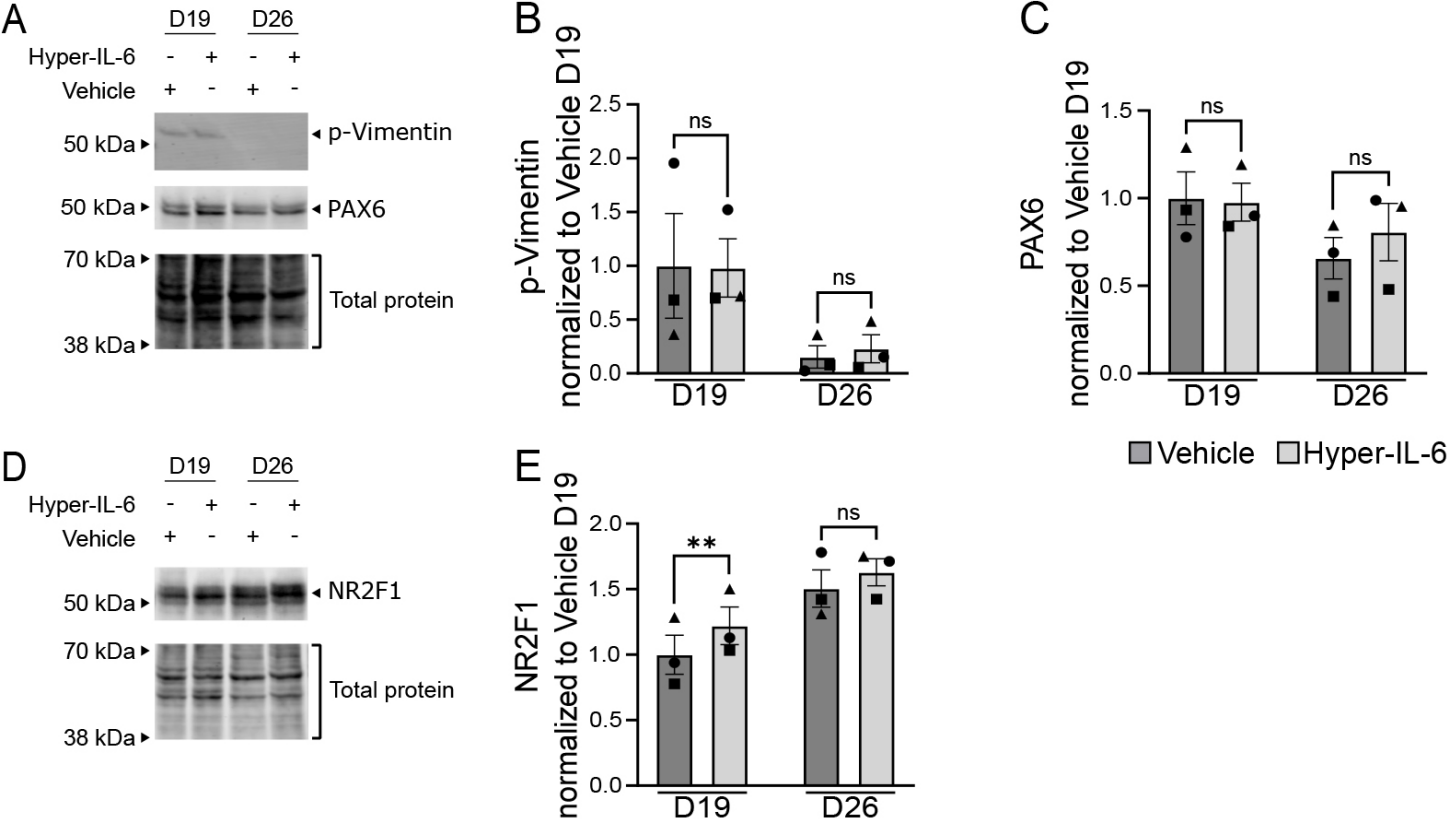
**Hyper-IL-6 treatment does not affect proliferation of iPSC-derived NPCs.** (A) Representative western blot of p-vimentin (top), PAX6 (middle) and total protein (bottom) in iPSC-derived NPCs after 5 days of treatment with Hyper-IL-6 as well as with vehicle. (B) Quantification of signal intensity of p-vimentin relative to total protein from A. (C) Quantification of signal intensity of PAX6 relative to total protein from A. (D) Representative western blot of NR2F1 (top) and total protein (bottom) in iPSC-derived NPCs after 5 days of treatment with Hyper-IL-6 as well as with vehicle. (E) Quantification of signal intensity of NR2F1 relative to total protein stain from D. For B, C and E, each dot represents an individual protein sample from consecutive differentiation batches of iPSC-derived NPCs (*n*=3 batches). Data are mean±s.e.m. Comparisons were analyzed using paired two-tailed Student's *t*-test separately for D19 and D26: ns, *P*≥0.05; **P<0.01.

In a mouse model of MIA, IL-6 increases the proliferation of NPCs and upregulates the expression of PAX6, a marker of neocortical NPCs (Ben-Reuven and Reiner, 2021). To assess whether a similar effect can be shown in human iPSC-derived NPCs, we analyzed the expression of p-vimentin, a marker of proliferative NPCs, and PAX6 upon exposure to Hyper-IL-6 (Fig. 4A-C) following the previously described treatment paradigm (Fig. 3A). We found no changes in p-vimentin and PAX6 levels between control and Hyper-IL-6-treated samples (Fig. 4B,C, *P*≥0.05). Interestingly, NR2F1, a transcription factor that was found to be upregulated upon Hyper-IL-6 exposure in dorsal forebrain organoids (Sarieva et al., 2023), was also upregulated in the Hyper-IL-6-treated neural cultures at D19 (*P*=0.0075) but not at D26 (*P*≥0.05), compared to vehicle-treated controls (Fig. 4D,E), indicating that although cellular changes cannot be modeled in 2D cultures, the molecular responses to MIA are shared between the 2D and 3D models.

## DISCUSSION

MIA has been shown to increase the risk of neurodevelopmental disorders such as ASD or schizophrenia in the offspring (Atladóttir et al., 2010; Brown, 2012). Although the molecular mechanisms that mediate the effects of MIA on prenatal neurodevelopment are not fully understood, inflammatory mediators, including IL-6, have been shown to play a causal role (Smith et al., 2007; Ben-Reuven and Reiner, 2021). Here, we characterized responses of ReNCell CX cells, an immortalized line of human NPCs, and human iPSC-derived NPCs to IL-6 following activation of *cis*- and *trans-*signaling to establish a model that can be used to study the molecular changes downstream of MIA in a human 2D model.

ReNCell CX cells have been used to study the effects of environmental exposures on neurodevelopment at the cellular level (Kwan Cheung et al., 2022; Zhao et al., 2013). Similarly, cell lines generated from different brain regions were also subjected to a range of inflammatory mediators, including IL-6 (Johansson et al., 2008). The authors showed expression of IL6R in these cells (Johansson et al., 2008). In our study, we show that ReNCell CX cells expressed IL6ST. We, therefore, suggest that co-expression of both IL6R and IL6ST in ReNCell CX cells allowed the phosphorylation of STAT3 at Y705 upon both IL-6 and Hyper-IL-6 exposure. We observed that ReNCell CX cells can respond to the *cis* mode of IL-6 signaling, which is not reminiscent of the human developing neocortex in which IL6R is expressed exclusively in cells of non-neuroectodermal origin (Nowakowski et al., 2017; Sarieva et al., 2023). However, activation of the JAK/STAT3 signaling cascade did not result in changes in the proliferative capacity of the ReNCell CX cells. Previous reports also suggest that ReNCell CX cells do not generate electrophysiologically active neurons upon differentiating conditions (Donato et al., 2007) and do not, therefore, recapitulate neurogenesis. Taking these results together, we suggest that ReNCell CX cells recapitulate some of the molecular, but not cellular, properties of NPCs in the developing human neocortex in the context of the IL-6 response and are, therefore, a limited tool to study gene expression changes following MIA induction *in vitro*.

Much progress in establishing human 2D and 3D models of human neurodevelopment has been made since the introduction of human iPSCs (Shi et al., 2012; Lancaster and Knoblich, 2014; Kadoshima et al., 2013). iPSCs can differentiate into cells of all three germ layers, with several protocols existing for the differentiation of NPCs (Shi et al., 2012; Reinhardt et al., 2013). In this study, we employed a human-specific 2D system using iPSC-derived NPCs and modeled MIA by administering Hyper-IL-6. Although we focused our proof-of-concept study on one cell line, future studies may investigate to which extent these findings are reproducible in other iPSC lines. We chose a chronic treatment paradigm (5 days) to account for neural differentiation occurring in these cells and the prolonged increase in cytokines in MIA *in vivo*. The iPSCs successfully differentiated into NPCs of forebrain identity and cultures activated the JAK/STAT3 pathway upon Hyper-IL-6 treatment. Together with previous results showing that iPSC-derived NPCs are not capable of *cis* IL-6 signaling (Couch et al., 2023), these results indicate that Hyper-IL-6 exposure can be used to model MIA in a 2D *in vitro* system with human-specific genetic background. In contrast to a mouse study of MIA (Ben-Reuven and Reiner, 2021), we did not see increased proliferation of NPCs upon chronic Hyper-IL-6 treatment, as evidenced by the lack of change in p-vimentin expression in western blotting. However, this approach may not be sensitive enough for mild changes in proliferation. Hence, other quantitative techniques, such as EdU incorporation, can be applied to validate our observations. Additionally, we found that the transcription factor NR2F1 was upregulated in Hyper-IL-6-treated cells, compared to that in vehicle control, on D19 of differentiation, which was not observed before in any MIA mouse models and could, therefore, represent a human-specific reaction to MIA as it was also observed in an organoid model (Sarieva et al., 2023).

Compared to our study modeling MIA with Hyper-IL-6 treatment in dorsal forebrain organoids, the 2D culture showed similar molecular responses (Sarieva et al., 2023). First, the expression pattern of IL6ST is similar between the two model systems and human neocortex at early midgestation (Sarieva et al., 2023). Second, both 2D culture and dorsal forebrain organoids phosphorylate STAT3 at Y705 in response to Hyper-IL-6. However, in the 3D model, we were able to show an over-representation of SOX2-positive NPCs (i.e. ventricular radial glia-like cells) upon Hyper-IL-6 treatment, whereas in 2D culture, we observed neither elevation of p-vimentin, a marker of proliferation in NPCs, nor increase in PAX6, a marker of neocortical NPCs. Similar to the 3D model, we found an elevated level of NR2F1 as a response to Hyper-IL-6 on D19 but not D26 of differentiation. Furthermore, in dorsal forebrain organoids, we found abnormalities in the laminar positioning of neurons in the putative cortical plate as a delayed effect of Hyper-IL-6 treatment (Sarieva et al., 2023). Although the underpinnings of this finding remain elusive, the cytoarchitectural properties of the 2D culture do not allow recapitulation of this effect. Therefore, the applicability of iPSC-derived 2D cultures is also limited to molecular and partially cellular levels, similar to ReNCell CX cells.

Together, both 2D *in vitro* approaches showed some similarity to neocortical development in humans. Specifically, both ReNCell CX cells and iPSC-derived NPCs were capable of eliciting phosphorylation of STAT3 upon Hyper-IL-6 treatment. Although the activation of the IL-6-dependent signaling pathway is robust with both approaches, the outcomes differ at the cellular level between the two 2D culture models as well as compared to the human 3D *in vitro* model and the mouse models of MIA (Ben-Reuven and Reiner, 2021; Sarieva et al., 2023). These discrepancies may stem from several differences that distinguish the models. First, the method of generation of the ReNCell CX cells by the introduction of c-Myc oncogene likely affects their expression profile and neurogenic properties and makes them an inappropriate model system for investigating the effects of MIA on the highly dynamic process that is neurodevelopment. Second, the differences in cytoarchitecture and cellular microenvironment between 2D and 3D models likely affect non-cell-autonomous mechanisms, including proliferation. Last, the differences between *in vitro* and *in vivo* studies go beyond the differences between two and three dimensions and include differences in the quality of the response (length and strength of exposure, types of cytokines increased) and cellular composition. Namely, both 2D and 3D models typically lack cells of non-neuroectodermal origin, such as microglia. In the context of MIA, these brain immune cells are crucial because, during human neocortical development, microglial precursors have already migrated to the ventricular zone by postconceptional week 4.5 (Monier et al., 2006, 2007) and are known to contribute to MIA (Cunningham et al., 2013). Nevertheless, using the engineered protein Hyper-IL-6 allowed us to recapitulate at least some of the secretory functions of microglia (Couch et al., 2023). In summary, here we describe to which extent the different 2D human neural cell culture models can be used to model MIA *in vitro*. Although we acknowledge the limitations of the iPSC-derived NPCs for modeling the effects of MIA on human neurodevelopment, we believe that they can still be useful to investigate the molecular effects of IL-6 in a human-specific context.

## MATERIALS AND METHODS

### ReNCell CX cell culturing and IL-6/Hyper-IL-6 treatment

ReNCell CX cells (Millipore, SCC007) were cultured following the manufacturer's instructions in ReNCell™ NSC Maintenance Medium (Millipore, SCM005) supplemented with 20 ng/ml FGF2 (Peprotech, 100-18B) and 20 ng/ml EGF (Sigma-Aldrich, GF144) on laminin (Merck, CC095)-coated six-well plates. The cultures were maintained in standard conditions (5% CO_2_, 37°C). The medium was changed every other day, and subculturing was performed every 3-4 days using Accutase (Merck, A6964). The cultures were treated with either IL-6 (35.25 ng/ml, 1.69 nM, R&D Systems, 7270-IL-025) or Hyper-IL-6 (100 ng/ml, 1.69 nM, R&D Systems, 8954-SR-025) diluted in 0.1% bovine serum albumin (BSA; AppliChem, A0850) in PBS (also used as vehicle control) for 1.5 h. For EdU experiments, half-volume media change was performed with the complete medium containing vehicle, IL-6, or Hyper-IL-6 and EdU (final concentration 10 µM), and cells were incubated until the termination of the experiment.

### iPSC culturing and neural differentiation

BIONi010-C iPSCs were obtained from the European Bank for induced pluripotent Stem Cells (EBiSC), cultured in standard conditions in E8 flex medium (Gibco, A2858501) and passaged in colonies using Gentle Dissociation Reagent (STEMCELL Technologies, 07174) onto human embryonic stem cell-qualified growth factor-reduced Matrigel-coated (Corning, 354277) plates. The routine quality checks were performed as described previously (Sarieva et al., 2023). iPSCs were differentiated towards neocortical neurons following a published protocol with minor adaptations (Shi et al., 2012). For neural induction, 70-80% confluent iPSCs were dissociated into single cells using Accutase, and plated on Matrigel-coated (Corning, 354234) plates in an E8 flex medium supplemented with 2 µM RI thiazovivin (Sigma-Aldrich, 420220) at a seeding density of 2.5×10^6^ cells/well in a six-well plate. The next day, differentiation was initiated with fully confluent wells through media change with neural induction (NI) medium [N2:B27 (Tables S1 and S2) 1:1 v:v, 10 µM SB431542 (Tocris, 1614), 1 µM Dorsomorphin (Tocris, 3093)]. NI medium was exchanged daily until D12, when cells were subcultured in colonies by applying Gentle Dissociation Reagent (STEMCELL Technologies, 07174). Cell clumps from every well were collected and distributed between three wells of poly-L-ornithine hydrobromide (Sigma-Aldrich, P4957)/laminin-coated six-well plates in NI medium. The following day, the medium was changed to neural maintenance (NM) medium (N2:B27 1:1 v:v). When rosette structures started to appear, 20 ng/ml FGF2 was added to the cells for 2-4 days to promote the expansion of NPCs. On D19, cells were passaged as described for D12, except that the seeding of cells was performed in NM medium. The next day, the medium was exchanged and, from thereon, half of the NM medium was changed every other day until D26 of differentiation.

### IL-6/Hyper-IL-6 treatment in iPSC-derived NPCs

The cultures were treated with Hyper-IL-6 diluted in 0.1% BSA in PBS (also used as vehicle control). At the start of treatment, a complete media change was performed with either NI (D14-D19) or NM (D21-D26) medium containing Hyper-IL-6/vehicle to result in a final concentration of 25 ng/ml (422.6 pM) Hyper-IL-6. The treatment was maintained by daily half-volume media change until the termination of the experiment.

### Immunocytochemistry and CLICK chemistry-based EdU detection

The cells were fixed in 4% paraformaldehyde in PBS (Morphisto, 10303.0025) for 8 min at room temperature (RT) and subsequently washed three times with PBS. Permeabilization was performed using 0.5% Triton X-100 in PBS for 10 min, and cells were washed once again with PBS.

Where appropriate, CLICK chemistry-based EdU detection was performed at this stage with a Click-iT EdU Cell Proliferation Kit (Thermo Fisher Scientific, C10338) following the manufacturer's instructions. Briefly, after permeabilization, the cells were washed three times with 3% BSA in PBS and incubated with the CLICK reaction cocktail for 30 min at RT. The cells were washed once in 3% BSA in PBS and immunocytochemistry was performed as follows.

Non-specific sites were blocked with 10% donkey serum (Abcam, ab7475) in PBS for 1 h at RT before cells were incubated with primary antibodies (Table S3) diluted in blocking solution overnight at 4°C. Cells were washed three times with PBS and incubated with secondary antibodies (Table S4) diluted in the blocking solution for 1 h at RT (protected from light). After antibody removal, cells were washed two times with PBS and counterstained for 5 min with 1:5000 4′,6-diamidino-2-phenylindole (DAPI; Thermo Fisher Scientific, D1306) in PBS or for 10 min with 2 µM DRAQ5 (Thermo Fisher Scientific, 62251) in PBS. Coverslips were mounted onto glass slides with ProLong™ Gold Antifade Mountant medium (Thermo Fisher Scientific, P36930).

Images were acquired with Leica SP8 and Olympus FV3000RS confocal microscopes with appropriate software and processed with ImageJ. For better visualization, the brightness of images was adjusted and the ‘Despeckle’ function in ImageJ software was applied where appropriate.

### Protein electrophoresis and western blotting

Whole-cell protein extraction was performed by collecting the cells in 1× RIPA buffer (Cell Signaling Technology, 9806) supplemented with 1× Halt protease and phosphatase inhibitor (Thermo Fisher Scientific, 78443) on ice. Samples were incubated for 1 h on a rotating wheel at 4°C. Next, samples of iPSC-derived neural cultures were sonicated with a Bandelin SONOPULS mini20 sonicator. All samples were centrifuged at 4°C and 14000 ***g*** for 30 min, and supernatant was collected. Protein concentration was quantified with a Pierce BCA Protein Assay Kit (Thermo Fisher Scientific, 23227).

Proteins were denatured by boiling with Protein Sample Loading Buffer (Li-COR, 928-40004) at 95°C for 5 min. Then, 10 or 15 µg of protein was loaded in 4-15% Mini-PROTEAN TGX Precast Gels (Bio-Rad, 4561086) and run with Tris (25 mM)-Glycine (192 mM) running buffer with 0.1% SDS (Bio-Rad, 1610732). Proteins were transferred on PVDF membranes (Bio-Rad, 1620264) by semi-dry transfer using Tris (25 mM)-Glycine (192 mM), 0.1% SDS and 10% methanol transfer buffer with a Trans-Blot Turbo system (Bio-Rad). For quantification of total protein, a Revert 700 Total Protein Stain Kit for Western Blot Normalization (Li-COR, 926-11010) was used following the manufacturer's instructions, and membranes were imaged with an Odyssey Imaging System (Li-COR). Following destaining, membranes were blocked using Intercept [Tris-buffered saline (TBS)-based] Blocking Buffer (Li-COR, 927-60001) for 1 h at RT and incubated with primary antibodies (Table S5) diluted in Intercept (TBS-based) Antibody Diluent (Li-COR, 927-65001) overnight at 4°C. Membranes were washed three times for 5 min with TBS with 0.1% Tween 20 (TBST) before incubation for 1 h at RT with secondary antibodies (Table S6) diluted in Intercept Antibody Diluent. Membranes were washed again three times with TBST (5 min each) and rinsed once with TBS to remove residual Tween 20. Immunodetection was performed with the Odyssey Imaging System, and images were analyzed using Image Studio Lite v.5.2 (Li-COR) software.

### Statistical analysis and visualization

Prism v.9.5 (GraphPad Software) was used for statistical analysis of the data. Data are presented as mean±s.e.m. The tests used for statistical analysis are described in the figure captions and the text. *P*<0.05 was considered statistically significant.

## Supplementary Material

10.1242/dmm.050306_sup1 Supplementary information
