# Supplementary material for: Pluripotent stem cell-derived neural progenitor cells can be used to model effects of IL-6 on human neurodevelopment
Source: Dis Model Mech. 2023 Nov 3;16(11):dmm050306. doi: 10.1242/dmm.050306 (PMC10629675; doi:10.1242/dmm.050306)
Supplement: Supplementary information [file dmm-16-050306-s1.pdf]

Table S1. Composition of the N2 medium

Compound	Cat. No., manufacturer	Final concentration
**DMEM-F12/Glutamax**	31331093, Gibco	-
**N2 supplement**	17502048, Gibco	1x
**Pen/Strep**	P0781, Sigma-Aldrich	1x
**MEM Non-essential Amino Acid Solution**	M7145, Sigma-Aldrich	1x
**2-Mercaptoethanol (55 mM)**	21985023, Gibco	100 µM
**Insulin (10 mg/ml)**	I9278, Sigma-Aldrich	5 µg/ml

Table S2. Composition of the B27 medium

Compound	Cat. No., manufacturer	Final concentration
**Neurobasal medium**	21103049, Gibco	-
**B27 supplement**	17504044, Gibco	1x
**Glutamax**	35050038, Gibco	200 mM
**Pen/Strep**	P0781, Sigma-Aldrich	1x

Table S3. Primary antibodies for immunofluorescence analysis

Target protein	Cat. No., manufacturer	Lot no.	Clonality, host species	Dilution factor
**FOXG1**	ab18259, Abcam	GR3297871-1	Polyclonal, rabbit	1:500 /1:250
**IL6ST**	AF-228-NA, R&D Systems	QX0518061	Polyclonal, goat	1:100
**SOX2**	AF2018, R&D Systems	KOY0521031, KOY0622061	Polyclonal, goat	1:500
**TBR2**	HPA028896, Atlas Antibodies	000016827	Polyclonal, rabbit	1:100
**Tuj1**	AMAb91394, Atlas Antibodies	MAB-03204	Monoclonal, mouse	1:1000/1:500

Disease Models & Mechanisms • Supplementary information

Table S4. Secondary antibodies for immunofluorescence analysis

Target species	Cat. No., manufacturer	Lot no.	Host species	Dilution factor	Conjugated dye
**Goat**	Ab150130, Abcam	GR3220578-5	Donkey	1:2000	Alexa Fluor 555
**Mouse**	A-2120, Thermofisher	1110071	Donkey	1:2000	Alexa Fluor 488
**Rabbit**	A-31573, Thermofisher	2083195	Donkey	1:2000	Alexa Fluor 647

Table S5. Primary antibodies for Western blotting

Target protein	Cat. No., manufacturer	Lot No.	Clonality, host species	Dilution factor
**NR2F1**	ab181137, Abcam	GR3375241-3	Monoclonal, rabbit	1:1000
**STAT3**	9139, Cell Signaling Technology	12, 16	Monoclonal, mouse	1:1000
**p-Y705-STAT3**	9145, Cell Signaling Technology	43	Monoclonal, rabbit	1:1000
**p-Vimentin**	D076-3, MBL	038	Monoclonal, mouse	1:1000
**PAX6**	901302, Biolegend	B354381	Polyclonal, rabbit	1:2000

Table S6. Secondary antibodies for Western blotting

Target species	Cat. No., manufacturer	Lot no.	Host species	Dilution factor	Conjugated Dye
**Mouse**	926-68070, Li-COR	D00115-03	Goat	1:15000	IRDye® 680 RD
**Rabbit**	926-32211, Li-COR	C91211-03	Goat	1:15000	IRDye® 800RD

Disease Models & Mechanisms • Supplementary information
